# Mesenchymal stem cells reduce the genotoxic effect of lead acetate in the testis of male rats and induce testicular cellular proliferation indicated by 16S rRNA sequence, increase the proliferation marker Ki-67 and a reduction in the apoptosis marker caspase-3

**DOI:** 10.1186/s40659-025-00614-5

**Published:** 2025-05-27

**Authors:** Mohamed Allam, Yahia A. Amin, Samer S. Fouad, Rana A. Ali, Mariam A. Fawy, Maha Abd-El Baki Ahmed, Rana Toghan, Lobna A. Ali

**Affiliations:** 1 Department of Zoology, Faculty of Science, Luxor University, Luxor, Egypt; 2 Department of Theriogenology, Faculty of Veterinary Medicine, Aswan University, Aswan, 81528 Egypt; 3 Veterinary Clinical Pathology, Qena University Hospital, South Valley University, Qena, Egypt; 4 Department of Zoology, Faculty of Science, South Valley University, Qena, 83523 Egypt; 5 Department of Anatomy, Faculty of Medicine, South Valley University, Qena, Egypt; 6 Department of Physiology, Faculty of Medicine, South Valley University, Qena, Egypt; 7 Cell Biology and Histochemistry, Department of Zoology, Faculty of Science, South Valley University, Qena, Egypt

**Keywords:** Reproductive toxicity, Testis, Lead acetate, Mesenchymal stem cells, Caspase 3, Ki-67, 16S rRNA, Genetic distances

## Abstract

**Background:**

Lead is a ubiquitous environmental and industrial pollutant with worldwide health problems. Lead acetate toxicity induces both genotoxic effects and apoptosis. The present study aimed to investigate the usage of mesenchymal stem cells (MSCs) in the treatment of the genotoxic effect of lead acetate (LA) in the testis and its effect on the expression of the apoptosis marker caspase-3 and the proliferation marker Ki-67 in the injured testicular tissue.

**Methods:**

Twenty-one adult male rats were used in this investigation (7 rats/group). Group I received saline and served as the control group (ctrl group); Group II received lead acetate (100 mg/kg) and was called the LA group; Group III received both lead acetate (100 mg/kg) and MSCs (1 × 10^6^ cells/rat) and was called the LA-MSCs group. Body and testis weight, plus semen analysis, were performed in all groups. Reproductive hormones, serotonin, and cortisol were determined in sera. Additionally, oxidative/antioxidative status and lead acetate-induced genetic variations were investigated. Immunohistochemical staining for the proliferation marker Ki-67 and the apoptosis marker caspase-3 was also performed.

**Results:**

revealed that the weight of the body and testis and semen parameters (sperm count, viability, and motility) of the LA group exhibited significant reduction compared to the Ctrl and the LA-MSCs group. In addition, the LA group showed reproductive hormonal imbalance and an increase in oxidative stress biomarkers compared to the LA-MSCs group that showed a significant improvement in these parameters. Compared to the ctrl group, the LA group showed a highly genetic distance value (0.0031), while the LA-MSCs group showed a low genetic distance value (0.0019). This illustrated that the LA-MSCs group exhibited reduced genetic variation induced by LA compared to the LA group. Histological evaluation indicated the presence of severe diffuse degeneration and necrosis in the spermatocytes in the LA group compared to the control one, while co-treatment by MSCs induced significant reduction in these degenerative changes. Immunohistochemical investigation revealed increased expression of the caspase-3 antibody in the testicular tissue of the LA group, while it is significantly decreased in the LA- MSCs group. In contrast, the KI67 antibody revealed a significant decrease in its expression in the LA group, while it was significantly increased in the LA-MSCs group after treatment by MSCs.

**Conclusions:**

It can be concluded that the MSCs are a potential therapeutic for the treatment of testicular dysfunction induced by LA through the reduction of oxidative stress, genotoxic effect, and apoptosis marker caspase-3, and an increase in the proliferation marker Ki-67 in the testicular tissue associated with restoration of hormonal imbalance.

## Introduction

The rapid growth of contemporary industry has coincided with a sharp rise in heavy metal pollution of the environment in recent years. Human activity frequently causes the high levels of heavy metals in animal feed and feed products, whether through industrial production, agriculture, or unintentional or intentional usage [1]. Lead (Pb) is one of these metals, and its levels in both urban and periurban areas have significantly increased in recent years [2]. Paints, eye cosmetics, gasoline, enamels, and water pipes are important commercial products that contain this harmful material [3]. Lead causes a wide spectrum of physiological and biochemical dysfunctions and has a profoundly complex effect on the health of both humans and animals [4]. For examples, the neurological, hematological, gastrointestinal, cardiovascular, renal and reproductive systems are among the many bodily systems that are essentially impacted by this cumulative toxicant after being consumed, inhaled, or absorbed via the skin [5–8].

The precise mechanism of action of lead, a strong neurotoxic toxin, has not been fully understood despite decades of research and requires further clarification. The induction of apoptosis is one among the most proposed mechanisms of lead [9]. Because lead is a multi-target toxicant, it disrupts the delicate pro- and antioxidant balance in human cells by producing excessive amounts of reactive free radicals [5].

To lessen the symptoms of lead toxicity, a number of antioxidant strategies have recently been put forth [10]. A recent research has demonstrated the antioxidant properties of several natural products against a wide range of harmful metals [11].

In the male reproductive system, the pituitary–testicular axis is the primary site of major unfavorable lead reactions, which can result in morphological changes and a reduction in sperm count, ultimately leading to infertility [12]. Low androgen levels, increased aberrant sperm morphology, degeneration of spermatogonia and spermatocytes, necrosis of spermatogonia and Sertoli cells [13], and lower sperm quality [14], have all been linked to lead exposure. Lead inhibits the activities of the steroidogenic enzymes, which hinders spermatogenesis and Leydig cell steroidogenesis [15]. Follicle stimulating hormone (FSH), luteinizing hormone (LH), and testosterone production are all affected that may reach to their breakdown [16, 17]. Lead changes the maturation of sperm via affecting the epididymis [18].

Male reproductive impairment is caused by the toxic effects of lead, which are deposited in the testis, vas deferens, seminal vesicle, and ejaculate. Male reproductive failure linked to lead exposure has been linked to two mechanisms: apoptosis and testicular oxidative stress. According to previous report, lead raises the expression of the caspase-3 protein in rats'testes [19]. Additionally, it has been shown to promote lipid peroxidation of cells and a decrease in antioxidant enzyme activity, which leads to an overabundance of reactive oxygen species (ROS) and oxidative stress [19, 20]. Excessive ROS production has been connected to sexual dysfunction. Overproduction of ROS damages sperm DNA, sperm function, testicular metabolism, and fertilization-related fusion processes, leading to infertility [21].

Mesenchymal stem cells have been proved to be effective in treatment of variant diseases such as lung injury, liver injury and kidney injury, and their mechanisms include the following aspects. First, they play an anti-apoptotic role, stimulate tissue repair, and produce nutritional healing factors. Both transforming growth factor (TGF-β) and hepatocyte growth factor (HGF) have the ability to repair liver damage and cirrhosis [22, 23]. Lung damage is positively impacted by Nrf2 (NF-E2-related factor 2) and Keap1 [24, 25]. The healing of acute kidney injury is significantly impacted by both epidermal growth factor (EGF) and insulin-like growth factor (IGF) [26, 27]. Second, it is possible to create exosomes, which work with cytokines and contain a variety of tiny molecules, including proteins and nucleic acids.

Despite the above mentioned deleterious effect of lead toxicity and the above mentioned pharmacological and therapeutic properties of stem cells, little research has been done on the potential protective function of stem cells against the negative effects of reproductive toxicity caused by lead acetate. Therefore, the present study aimed to determine whether MSCs can ameliorate Pb-induced reproductive damage through investigation of regenerative effect of MSCs on genetic variation induced by LA toxicity and its effect on the expression of the apoptosis marker caspase-3 and proliferation marker Ki-67 in the testicular tissue.

## Materials and methods

### Chemicals

Lead acetate was purchased from El-Gomhouria Company for Chemicals and Laboratory Supplies, located in Assiut, Egypt. Kits of Malondialdehyde (MDA), Superoxide dismutase (SOD), Catalase (CAT) and Glutathione peroxidase (GPx) were purchased from Biodiagnostic company, 29 El Tahrir, Ad Doqi, El Omraniya, Giza Governorate 3,750,164. The MSCs were purchased from Lab of Prof. Dr. Heba M. saad Eldien (Assiut University, Assiut, Egypt).

### Animals

Twenty one male Albino Wistar rats, weighing 180–200 g, were obtained from the Department of Zoology’s Animal House, Faculty of Science, South Valley University, Qena, Egypt. The animals were maintained at a controlled temperature of 24 ± 1 °C with a 12–12 h light–dark cycle, and were allowed free access to water and standard chow ad libitum.

### Experimental design

Twenty one male rats were randomly allocated into three groups (seven for each). The 1 st group received saline and served as the normal control group (ctrl group), while the 2nd group injected intraperitoneally with lead acetate (LA) (100 mg/kg body weight for seven days) and served as the standard model of acute LA-toxicity group [28] and called LA group. The 3rd group injected intraperitoneally with lead acetate (100 mg/kg b.w.t for seven days) and treated with a single dosage of mesenchymal stem cells (1 × 10^6^ cells/rat intravenous route) [29, 30] then left for 30 days and called LA-MSCs group.

### Determination of the body and testicular weights

A sensitive electronic weighing balance (Scout Pro, Ohaus Corporation, USA) was used to assess body weight every day. All of the rats were sacrificed at the end of the trial using an appropriate dosage of ethyl ether. After dissection, an absolute weight of the testis was measured using the same device during sacrifice. In addition, extraction testis tissues was performed followed by one was immediately placed on ice, and after that, it was placed in liquid nitrogen, where it was promptly frozen at − 80 °C until the tissue was homogenized for biochemical analysis. While the other one for a full of 24 h was fixed in 10% natural formalin. For histopathology, it was then stored in 70% ethyl alcohol. Blood was collected and serum was separated by centrifugation at 3000 rpm for 15 min and stored at (− 20 °C) for hormonal analysis.

### Sperm collections and assessments of semen parameters

Epididymal sperm counts was performed according to the previous research [31], was used to do epididymal sperm counts. This method involved chopping the caudal epididymis in 5 mL of 0.9% NaCl and thoroughly mixing each sample with a shaker for 10 min. For two minutes, the suspension of epididymal sperm was incubated at 20 °C. For the supernatant dilution (1:100), an alkaline solution including eosin and 35% formalin was utilized. Sperm were counted using a Neubauer hemocytometer under a 200 × light microscope. The count is reported in millions per milliliter of the epididymal solution. A high-magnification (400×) light microscope was used to calculate the percentage of total sperm motility [32, 33]. Evaluations of sperm count and morphology were conducted in compliance with WHO recommendations.

Regarding sperm motility, evaluation was classified as grade A (fast progressive sperms that swim quickly in a straight-forward direction), grade B (slow progressive sperms that move forward but in a haphazard line) and grade C (non-progressive sperms that can move their tails but can’t move forward).

### Determination of serum levels of reproductive hormones, serotonin and cortisol

Commercially available ELISA kits (supplied by Biomatik Co., Kitchener, Ontario N2 C 1 N6, Canada) were used for determination of serum hormone assays for follicle-stimulating hormone (FSH), testosterone, and gonadotropin-releasing hormone (GnRH) with catalogue numbers: EKL54319, EKF58719, EKF60674, respectively. ELISA kits for the detection of serotonin and cortisol were purchased from BioSource Co., with catalogue numbers MBS494156 and MBS843477, respectively.

### Preparation of tissue homogenate and oxidative analysis:

A portion of testis was homogenized by manual technique. Homogenates of the tissues were prepared in 1.0 ml of phosphate buffer per 100 mg of tissue. The samples were spun at maximum speed at 4 °C for the biochemical examination, and the supernatant was used. Tetramethoxypropane was used as an external standard in measurement of malondialdehyde (MDA) in accordance with the methodology of Ohkawa et al. [34]. The ability of superoxide dismutase (SOD) to prevent epinephrine from autoxidizing in an alkaline solution was used to measure its activity [35]. A colorimetric kit was used to assay catalase (CAT) activity in accordance with Lück’s [36]. Glutathione peroxidase (GPx) was determined according to Flohé and Günzler [37]. The supernatants from the tissue homogenates were used for colorimetric assays of (MDA, SOD, CAT & GPx in the tissues (using commercial kits supplied by Biodiagnostics, Egypt) with a spectrophotometer (Chem-7, Erba Diagnostics Mannheim GmbH, Germany).

### DNA extraction

By using the DNA extraction method of QIAamp DNA Mini kit (Qiagen, Hidden, Germany) DNA was isolated from the tissues following the manufacturer’s instructions.

### PCR amplification and sequencing

Primers that were previously described by Simon et al. [38] were used in the amplification of genomic DNA using a total volume of 40 μL. Including 1 μL of DNA, 1 μL of both forward and reverse primers, 17 μL of nuclease-free water, and 20 μL of master mix. In the PCR amplification process, an initial denaturation at 95 °C for three minutes was followed by thirty cycles of denaturation, annealing, and extension for one minute at 94 °C, 48 °C, and 72 °C, respectively. At 72 °C, cycling was finalized with a 10-min extension. Electrophoresis was used to separate and visualize the PCR products on an agarose gel stained with 1.5% ethidium bromide. Each group produced a single band from the PCR amplification. To obtain the accession numbers, the sequences were sent to GenBank/NCBI.

### Alignment and phylogenetic analysis

Sequence alignment was performed using MUSCLE [39] with default settings. Phylogenetic analyses among the three groups and *Rattus norvegicus* MZ782915.1from GenBank based on partial sequences of (16S rRNA) gene were performed with MEGA version 11.0.11 [40] using Neighbour Joining phylogenetic method. The bootstrap analysis was determined with 1000 replicates [41]. The sequence divergences were calculated using Kimura2-parameter distances [42].

### Tissue processing

Rats were slaughtered at the end of the study, and the left and right testes were removed, weighed, and prepared. Fixation in 10% Neutral Buffered Formalin, dehydration with increasing alcohol grades (two changes of 70%, 90%, and 100% alcohol), clearing in xylene, embedding in molten paraffin, and cooling to produce tissue blocks were all steps in the tissue processing procedure.

### Tissue sectioning and staining

Haematoxylin and Eosin (H&E) staining was applied to blocks of study tissues that had been cut into slices 5 μ thick. In order to stain, the following steps were taken: dewax in xylene, hydrate with decreasing alcohol grades (100%, 90%, and 70% alcohol) and distilled water, stain in hematoxylin, wash under running water, distinguish in 1% acid alcohol (1% HCl in 70% alcohol) for, blue in tap water for, rinse in distilled water, stain in eosin, rinse in distilled water, dehydrate with increasing alcohol grades (70%, 90%, and 100% alcohol), clear in xylene, and mount using Distrene polystyrene xylene (DPX).

### Histological score of Johnsen’s score (score)

According to Johnson scoring system, the spermatogenesis was evaluated with five sections per slide and 10 seminiferous tubules per field with a scale of 1–10 points [43, 44].

### Immunohistochemical investigation

The immunohistochemical staining procedures were done as the following: sections were dewaxed and immersed in a solution of 0.05 M citrate buffer, pH 6.8 for antigen retrieval. These sections were then treated with 0.3% (H2O2) and protein block. Then, sections were incubated with polyclonal anti-caspase 3 antibodies (Invitrogen, Cat# PA5-77,887, dilution 1/100), and monoclonal mouse of Ki67 antibody, Dako, USA M7248 and 1/100 dilution. After rinsing with phosphate buffered saline, they were incubated with a goat anti-rabbit secondary antibody (Cat# K4003, EnVision + ™ System Horseradish Peroxidase Labelled Polymer; Dako) for 30 min at room temperature. Slides were visualized with DAB kit and eventually stained with Mayer's hematoxylin as a counter stain. The immunolabelling indices of both caspase 3 and Ki67 were presented as a percentage of positive expression in a total 1000 cells per 8 HPF.

### Statistical analysis

Means ± Standard deviation of means (Mean ± S.D) was used to express the degree of results variability. A one-way ANOVA analysis of variance was used to assess the data statistically (the Prism pad computer software, USA) then the Newman-keuls T-test, and to assess for treatment differences, the least significant difference (L.S.D.) was utilized. When the P-value of the results is < (0.001), they are considered statistically significant.

## Results

### Body and testicular weights

Initially, all rats had almost the same body weight at the start of the experiment. However, by the end of the experiment, both the LA group and the LA-MSCs group experienced a significant decrease in body weight compared to the control group (P < 0.001, P < 0.01), respectively. Surprisingly, there was a significant increase in the weight of LA-MSCs group when compared to LA group (P < 0.01) (Table 1).

**Table 1:** The effect of lead acetate and mesenchymal stem cells on body and testicular weight of adult male albino rats

Items	Ctrl group	LA group	LA-MSCs group
Body weight (gram)	190.5 ± 6.89	152.7 ± 6.19 ^a−^	165.7 ± 7.42 ^a−b+^
Weight of testes (gram)	1.767 ± 0.14	1.108 ± 0.22 ^a−^	1.817 ± 0.13 ^b+^

All Values are mean ± S.D (n = 7)

*Ctrl group*: control group, *LA group* Lead acetate group, *LA-MSCs group* lead acetate co-treated with mesenchymal stem cells group

+ Significant increase at (p < 0.05). a → significantly different from control group

−Significant decrease at(p < 0.05). b → significantly different from Lead group

Regarding testicular weight, LA group exhibits a significant reduction in comparison to both control and LA-MSCs groups (P < 0.001). In contrast, there was no significant difference between control and LA-MSCs group (Table 1).

### Sperms count, viability and motility

As shown in Table 2, the sperm count, and viability were significantly decreased in LA group (P < 0.001) in comparison to the control and LA-MSCs group (P < 0.05). Interestingly LA- MSCs group shows a significant increase when compared to LA group (P < 0.01). In control group, no pus cells were found in semen, but LA group exhibits a significant increase in comparison to control group (P < 0.001), while LA-MSCs group shows a considerable decrease when compared to LA group (P < 0.001).

**Table 2:** Sperm count, viability and motility of the control group, LA group and LA-MSCs group of adult male albino rats

Parameters/groups		Ctrl group	LA group	LA-MSCs group
Sperm count × 10^6^		148.7 ± 33.90	43.00 ± 27.09^a−^	109.5 ± 11.22^b+^
Sperm dead %		23.33 ± 12.11	71.67 ± 36.70^a+^	61.00 ± 14.00^a+^
Sperm alive %		76.67 ± 12.11	11.67 ± 12.11^a−^	39.00 ± 14.00^a−b+^
Pus in semen		0.0 ± 0.0	7.833 ± 1.94^a+^	1.333 ± 0.82^b−^
Alive motility	A	68.33 ± 8.17	00.00 ± 00.00^a−^	31.83 ± 15.84^a−b+^
	B	20.83 ± 5.85	6.67 ± 16.33	16.33 ± 19.04
	C	10.83 ± 5.85	60.00 ± 48.99	51.83 ± 28.74

All Values are mean ± S.D (n = 7)

*Ctrl group* control group; *LA group* Lead acetate group, *LA-MSCs group* lead acetate co-treated with mesenchymal stem cells group

+ Significant increase at (p < 0.05). a → significantly different from control group

−Significant decrease at (p < 0.05). b → significantly different from Lead group

Regarding sperm motility: grade A was absent in LA group, shows a significant increase in LA-MSCs group (P < 0.001). For grade B, both LA and LA-MSCs groups show a non-significant differences compared to the control group. For grade C, no significant difference was observed among groups.

### Serum reproductive hormones, serotonin and cortisol

**Fig. 1:**
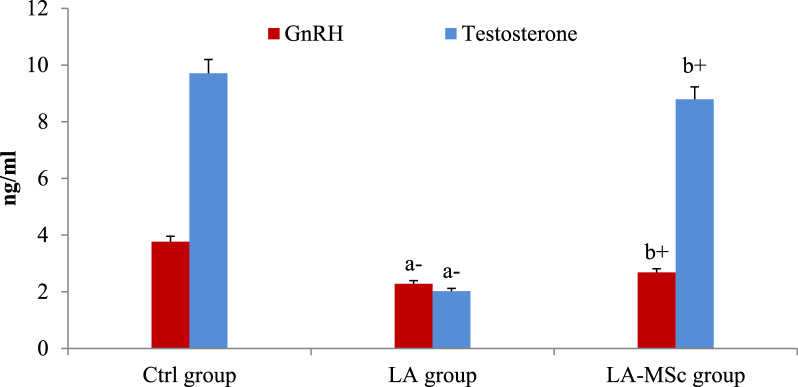
Serum levels of gonadotropin-releasing hormone (GnRH), and testosterone in the control group, LA group, and LA-MSCs group of adult male albino rats. Ctrl group: control group; LA group: Lead acetate group and LA- MSCs group: lead acetate co-treated with mesenchymal stem cells group. + Significant increase at (p < 0.05). a → significantly different from control group.—Significant decrease at (p < 0.05). b → significantly different from Lead group. All Values are mean ± S.D (n = 7)

**Fig. 2:**
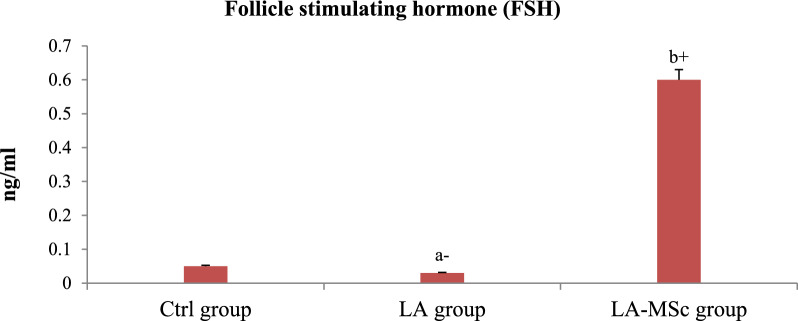
Serum levels of follicle-stimulating hormone (FSH) in the control group, LA group, and LA-MSCs group of adult male albino rats. Ctrl group: control group; LA group: Lead acetate group and LA-MSCs group: lead acetate co-treated with mesenchymal stem cells group. + Significant increase at (p < 0.05). a → significantly different from control group.—Significant decrease at (p < 0.05). b → significantly different from Lead group. All Values are mean ± S.D (n = 7)

**Fig. 3:**
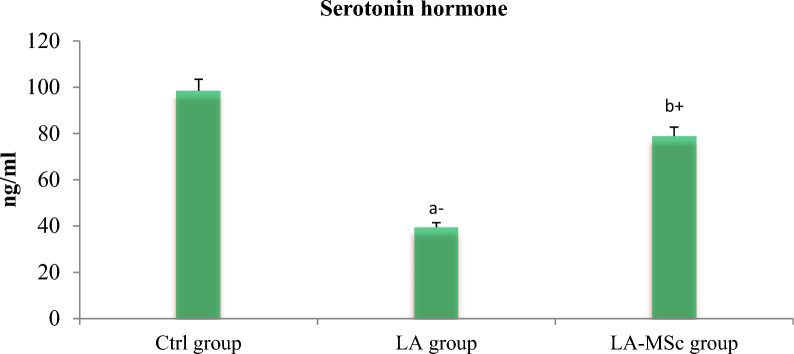
Serum levels of serotonin in the control group, LA group, and LA-MSCs group of adult male albino rats. Ctrl group: control group; LA group: Lead acetate group and LA-MSCs group: lead acetate co-treated with mesenchymal stem cells group. + Significant increase at (p < 0.05). a → significantly different from control group.—Significant decrease at (p < 0.05). b → significantly different from Lead group. All Values are mean ± S.D (n = 7)

**Fig. 4:**
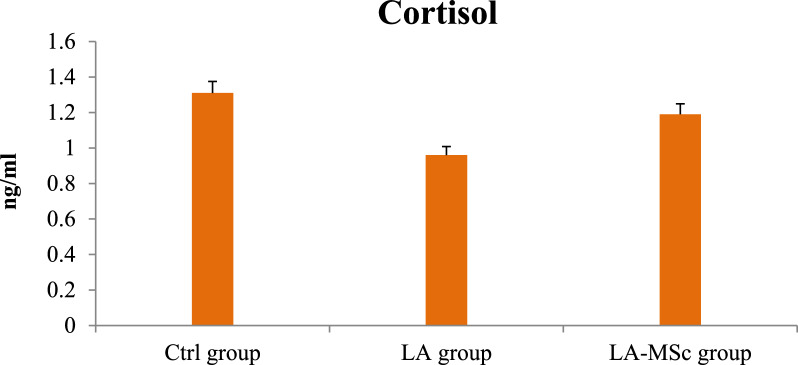
Serum levels of cortisol in the control group, LA group, and LA-MSCs group of adult male albino rats. Ctrl group: control group; LA group: Lead acetate group and LA-MSCs group: lead acetate co-treated with mesenchymal stem cells group. All Values are mean ± S.D (n = 7)

Table 3 shows the serum levels of the reproductive hormones, serotonin and cortisol in the tested group. Compared to the control group, serum levels of GnRH, testosterone, FSH, and serotonin were significantly decreased (P < 0.001) in the LA group (Figs. 1, 2 and 3). In contrast, the group of LA-MSCs showed significant increase in the all previously mentioned hormones compared to the LA group. Serum cortisol shows non-significant difference among groups (Fig. 4).

**Table 3:** Reproductive hormones, serotonin and cortisol in the serum of control group, LA group and LA-MSCs group of adult male albino rats

Items	Ctrl group	LA group	LA-MSc group
GnRH (ng\ml)	3.770 ± 0.21	2.283 ± 0.44^a−^	2.683 ± 0.23^b+^
Testosterone (ng\ml)	9.713 ± 0.67	2.027 ± 0.66^a−^	8.795 ± 0.68^b+^
FSH (ng\ml)	0.05 ± 0.10	0.03 ± 0.10^a−^	0.06 ± 0.10^b+^
Serotonin (ng\ml)	98.50 ± 5.32	39.50 ± 6.41^a−^	73.83 ± 12.88^b+^
Cortisol (ng\ml)	1.315 ± 0.44	0.9647 ± 0.12	1.199 ± 0.30

All Values are mean ± S.D (n = 7)

*Ctrl group* control group, *LA group* Lead acetate group, *LA-MSc group* lead acetate co-treated with mesenchymal stem cells group. *GnRH* Gonadotropin-releasing hormone, *FSH* follicle-stimulating hormone

+ Significant increase at (p < 0.05). a → significantly different from control group

−Significant decrease at (p < 0.05). b → significantly different from Lead group

### Evaluation of oxidative stress parameters

**Fig. 5:**
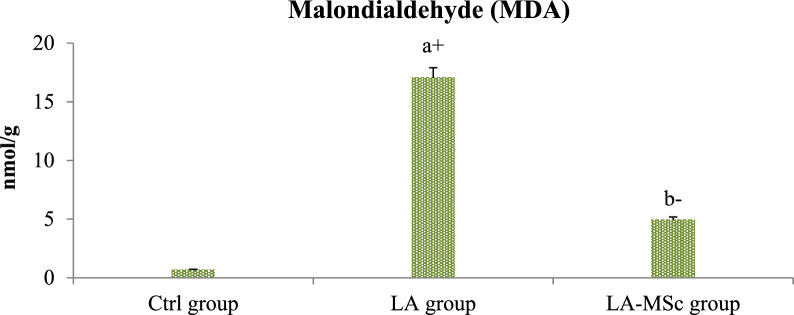
Levels of malondialdehyde (MDA) in the testicular tissue of the control group, LA group, and LA-MSCs group of adult male albino rats. Ctrl group: control group; LA group: Lead acetate group and LA-MSCs group: lead acetate co-treated with mesenchymal stem cells group. + Significant increase at (p < 0.05). a → significantly different from control group.—Significant decrease at (p < 0.05). b → significantly different from Lead group. All Values are mean ± S.D (n = 7)

**Fig. 6:**
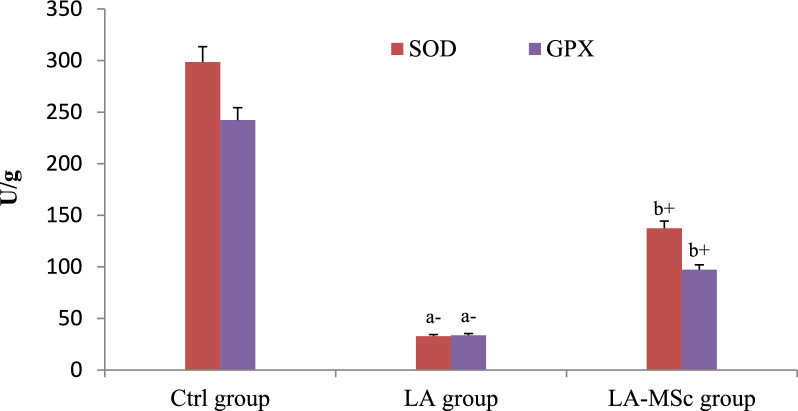
Levels of superoxide dismutase (SOD), and glutathione peroxidase (GPx) in the testicular tissue of the control group, LA group, and LA-MSCs group of adult male albino rats. Ctrl group: control group; LA group: Lead acetate group and LA-MSCc group: lead acetate co-treated with mesenchymal stem cells group. + Significant increase at (p < 0.05). a → significantly different from control group—Significant decrease at (p < 0.05). b → significantly different from Lead group. All Values are mean ± S.D (n = 7)

**Fig. 7:**
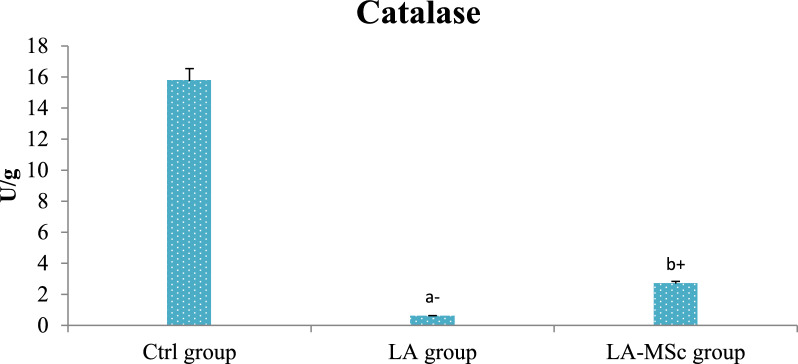
Levels of catalase (CAT) in the testicular tissue of the control group, LA group, and LA-MSCs group of adult male albino rats. Ctrl group: control group; LA group: Lead acetate group and LA-MSCc group: lead acetate co-treated with mesenchymal stem cells group. + Significant increase at (p < 0.05). a → significantly different from control group. - Significant decrease at (p < 0.05). b → significantly different from Lead group. All Values are mean ± S.D (n = 7)

As shown in Table 4, the level of MDA was significantly increased in LA group in comparison to control and LA-MSCs group (Fig. 5). In addition, there was a significant decrease in the levels of SOD, CAT and GPX in LA group in comparison to control group (P < 0.001) (Fig. 6 and 7). LA- MSCs group showed a significant increase in the three parameters in comparison to LA group.

**Table 4:** Oxidative stress biomarker and antioxidant profile in adult male albino rats of control group, LA group and LA-MSCs group

Items	Ctrl group	LA group	LA-MSCs group
MDA (nmol\ g)	0.69 ± 0.87	17.05 ± 0.83^a+^	4.943 ± 0.86^b−^
SOD (U/g)	298.5 ± 20.34	32.75 ± 9.78^a−^	137.0 ± 15.92^b+^
CAT (U/g)	15.75 ± 0.66	0.6005 ± 0.09^a−^	2.708 ± 1.04^b+^
GPX (U/g)	242.2 ± 27.51	33.62 ± 6.40^a−^	97.17 ± 19.35^b+^

All Values are mean ± S.D (n = 7)

*Ctrl group* control group, *LA group* Lead acetate group, *LA-MSCs group* lead acetate co-treated with mesenchymal stem cells group

*MDA* Malondialdehyde, *SOD* Superoxide dismutase, *CAT* catalase, *GPx* Glutathione peroxidase

+ Significant increase at (p < 0.05). a → significantly different from control group

−Significant decrease at (p < 0.05). b → significantly different from Lead group

### Genetic variation

In this investigation, Partial sequences of the mt16S rRNA gene in each of the three groups were identified. The fragment sizes ranged from 537 to 540 bp, with minimal size variation between groups. The16S rRNA’s partial nucleotide sequences of were add in the GenBank with accession numbers (OM570616- OM570618). The resulting concatenated alignment comprised 540 base pairs, of which 534 were sites that were conserved. The average A + T attribution was 60.8, surpassing than the C + G. Each group's sequence length, A + T and C + G contents, as well as their average, were shown in (Table 5).

**Table 5:** Accession numbers, nucleotide frequencies and their averages of (16S rRNA) gene in the control group, LA group and LA-MSCs group

Groups	Accession number	Base pair length	Nucleotide number %				A + T content (%)	C + G content (%)
			A%	T%	C %	G%		
Ctrl group	OM570616.1	537	33.34	27.37	19.55	19.74	60.71	39.29
LA group	OM570617.1	540	33.52	27.41	19.44	19.63	60.93	39.07
LA-MSCs group	OM570618.1	538	33.46	27.32	19.70	19.52	60.78	39.22
Average %	–	538	33.43	27.37	19.57	19.63	60.8	39.2

*Ctrl group* control group, *LA group* Lead acetate group, *LA-MSCs group*: lead acetate co-treated with mesenchymal stem cells group

Between the control and treated groups, the P-distances varied from 0.0000 to 0.0031. All groups combined had a distance value of 0.01%. The genetic effects of lead acetate were reflected in the highest P-distance (0.0031) between control group and LA group. However, there was a lower value (0.0019) between control group and LA-MSCs group (Table 6).

**Table 6:** Pairwise distances based on (16S rRNA) gene among the three groups and Rattus norvegicus MZ782915.1 from GenBank based on partial sequences of (16S rRNA) gene

Groups	OM570616.1 Ctrl group	MZ782915.1 Ctrl group	OM570617.1 LA group	OM570618.1 LA-MSCs group
OM570616.1—Control		0.0000	0.0031	0.0019
MZ782915.1—Control	0.0000		0.0031	0.0019
OM570617.1—Lead acetate	0.0056	0.0056		0.0037
OM570618.1—Lead acetate + stem cells	0.0019	0.0019	0.0075	

*Ctrl group* control group, *LA group* Lead acetate group, *LA-MSCs group* lead acetate co-treated with mesenchymal stem cells group

**Fig. 8:**
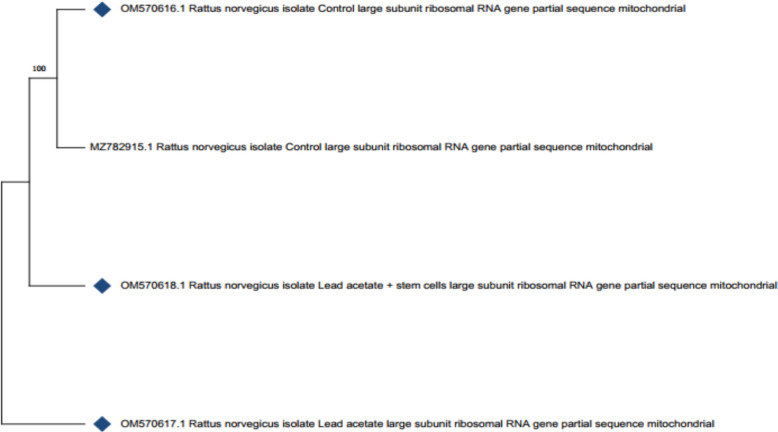
Phylogenetic tree using the Neighbour Joining method among the three groups and *Rattus norvegicus* MZ782915.1from GenBank based on partial sequences of (16S rRNA) gene

For every clade recovered by Neighbour Joining (NJ) clades, the study evaluated the level of support using bootstrap values. The phylogenetic analysis revealed that LA group formed a separate cluster, while the remaining (OM570616.1, MZ782915.1—Control and OM570618.1—Lead acetate + stem cells) found in one clad (Fig. 8).

### Histological investigation

**Fig. 9:**
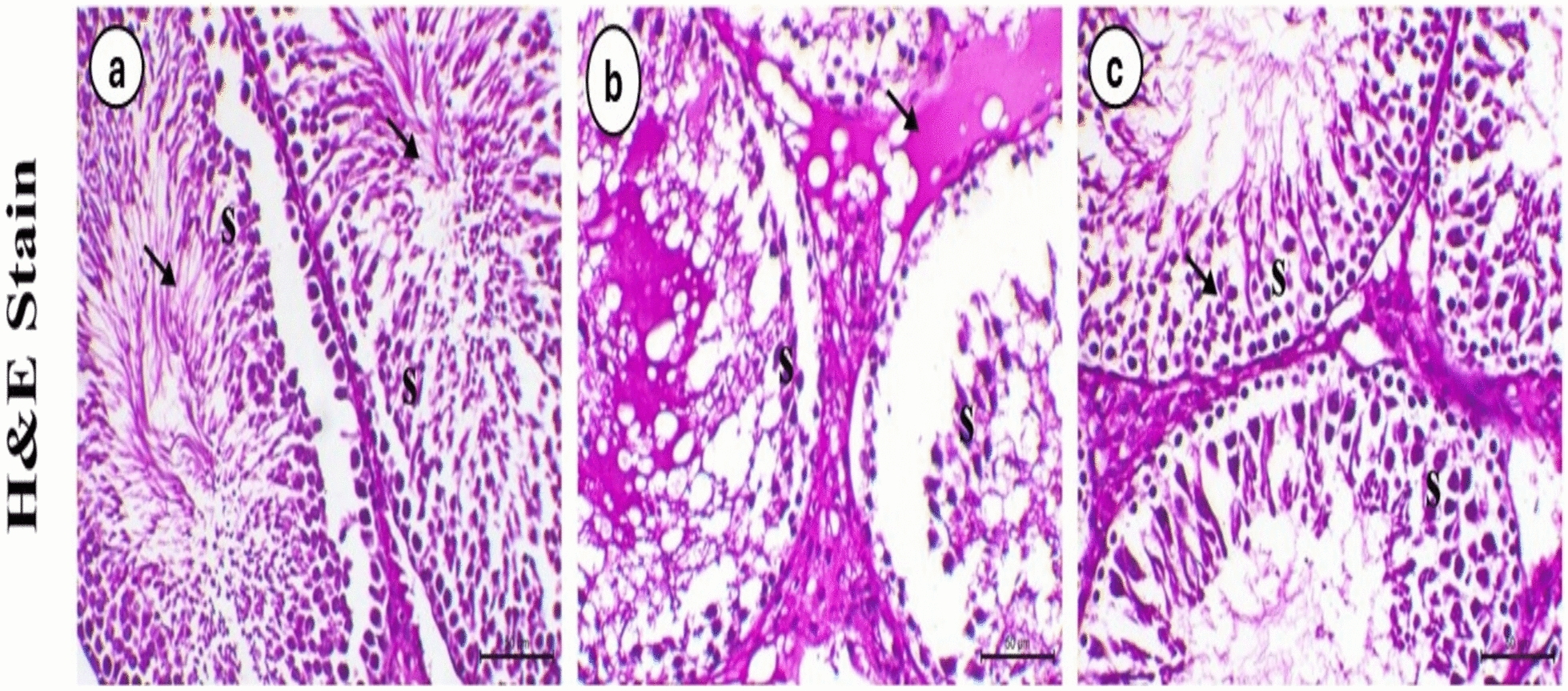
**a** Testis from the control group showing normal seminiferous tubules lined with intact spermatogenic cell layers (S) and abundant free sperm in the lumen (arrows). **b** Testis from the lead acetate group showing marked degenerative and necrotic changes in the seminiferous tubules (S), desquamation of necrotic cells, and interstitial edema (arrow). **c** Testis from the lead acetate + mesenchymal stem cells (MSCs) group showing a marked reduction in degenerative changes in spermatogenic cells (arrow), with the reappearance of sperm in the lumen. H&E staining, bar = 50 µm, × 200.

Figure 9 showed the pathological changes that occurred in the testis in the three tested groups using the same magnification. Testis of ctrl group showing normal seminiferous tubules lined with normal spermatogenic cell layers with presence of free sperm in the lumen. While, the testis of LA group showing marked degenerative and necrotic changes within the seminiferous tubules, desquamation of some necrotic cells and edema within the interstitial tissue. In addition, sever diffuse degeneration and necrosis in the spermatocytes was also observed compared to control one. After the treatment with MSCs, the testes are showing marked improvement of the degenerative changes within the spermatogenic cells with appearance of sperms within the lumen.

### Results of Johnsen score

Johnsen score was found 10 in the ctrl group that refers to maximum spermatogenesis activity, while it was 3.3 in LA group that refer to complete absence of spermatogenesis. Whereas the LA-MSCs group showed enhancement in the spermatogenic status as Johnsen score was found 8.6.

### The immunohistochemistry investigation

**Fig. 10:**
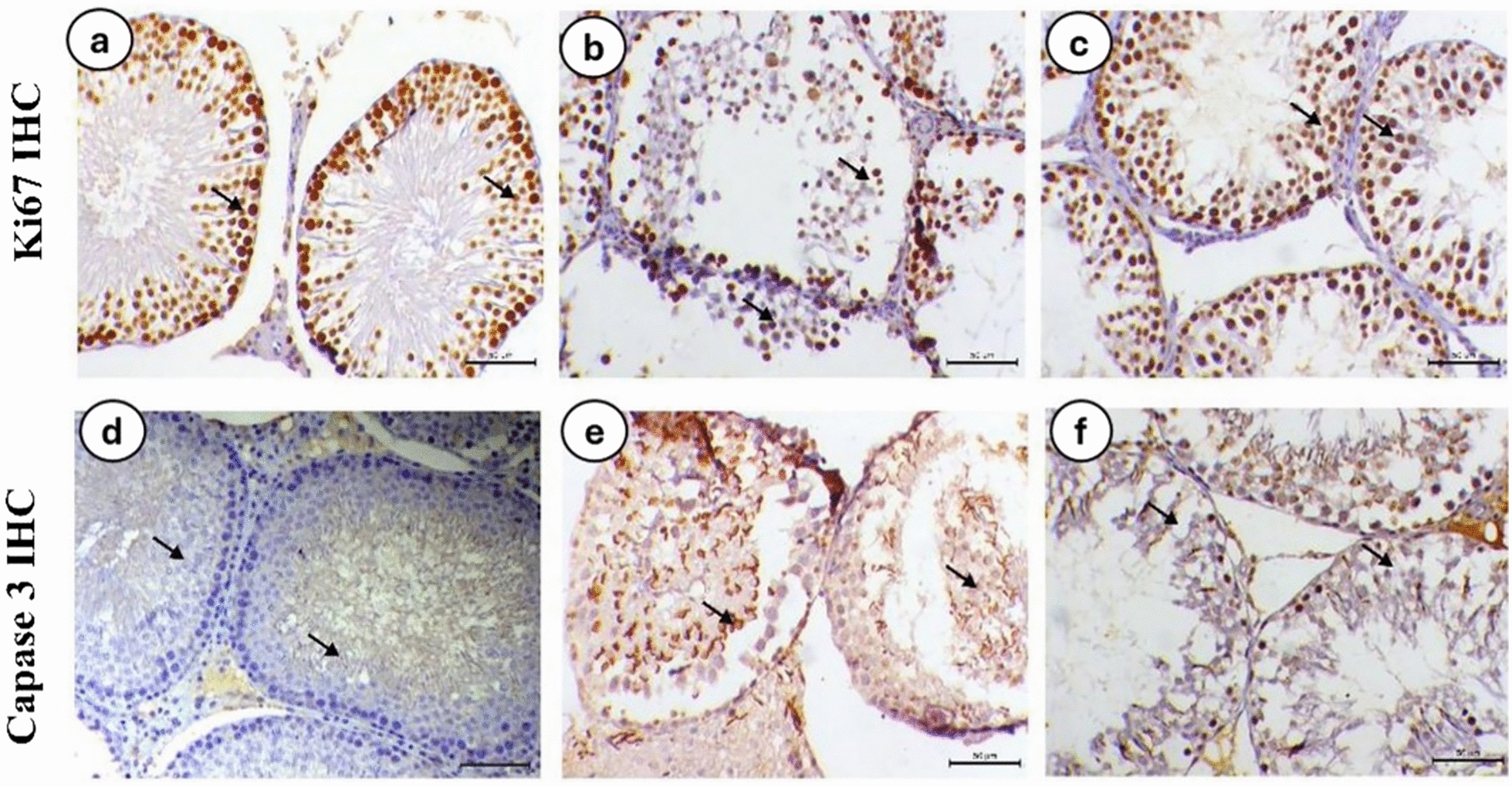
**a** Photomicrographs of testicular tissue from control animals immunostained with Ki67 antibody, showing strong nuclear expression of Ki67 in spermatogenic cells (arrows). **b** Photomicrographs of testicular tissue from the lead acetate group immunostained with Ki67 antibody, showing a significant decrease in Ki67 expression within spermatogenic cell layers (arrows). **c** Photomicrographs of testicular tissue from the lead acetate + mesenchymal stem cells (MSCs) group immunostained with Ki67 antibody, showing a marked increase in Ki67 expression in spermatogenic cells (arrows). **d** Photomicrographs of testicular tissue from control animals immunostained with caspase-3 antibody, showing mild cytoplasmic expression of caspase-3 in spermatogenic cells (arrows). **e** Photomicrographs of testicular tissue from the lead acetate group immunostained with caspase-3 antibody, showing significant increase in caspase-3 expression in spermatogenic cell layers (arrows indicating nuclear expression). **f** Photomicrographs of testicular tissue from the lead acetate + MSCs group immunostained with caspase-3 antibody, showing a marked decrease in caspase-3 expression in spermatogenic cells (arrows). Ki67 and caspase-3 IHC, bar = 50 µm, × 200.

The immunohistochemistry investigation of caspase3 and Ki67 were presented as a percentage of positive expression. Results revealed an obvious increase in the average percentage of Caspase3 antibody in the testicular tissue of LA group, while it is significantly decreased in the testis of rats treated with MSCs (LA-MSCs group). In contrast, statistics showed a normal KI67 percentage in the control group (2.2%), while the LA group showed a highly significant decreasing level of KI67 percentage (1.7%). The group of LA-MSCs showed a noticeable enhancement of KI67 percentage (2.9%) (Fig. 10).

## Discussion

Due to its pervasiveness in soil, water, and air, lead is a widely researched environmental pollutant that still poses a major risk to human health. It is commonly known that lead is hazardous to various systems, including the male reproductive system [45, 46].

In our study, significant decrease of the testes and body weights were observed in LA exposed rats compared to controls as well as MSCs treated group. This indicating that the general metabolic condition of LA treated animals suffers from disturbance in contrast to the animal received MSCs treatment which remains normal. Our result agreed with the previous studies that concerned with lead acetate toxicity [17, 19]. According to the latter research, rats given lead had considerably lower index weights for the testis, epididymis, and accessory sex glands than the control group.

In the current trial, the sperm motility investigation revealed that the lead-treated group experienced a disturbance in sperm motility, compared to the Ctrl and LA-MSC groups, which displayed normal sperm motility. This suggests that MSCs treatment had a positive effect on sperm motility compared to the untreated group. According to a recent study, the sperm motility test showed that the model + MSCs treatment group had significantly higher sperm motility than the LA toxic group. This finding supports the idea that MSCs have a reparative effect on the testicular tissue of mice and improve sperm motility [47]. These results also demonstrate that elevated lead levels are significantly negatively correlated with common semen characteristics and sperm function biomarkers [48, 49].

One of the most significant methods that were applied in variant studies as a method of evaluation of the fertility of animal and human is assessment of the reproductive hormones [50–52]. Compared with the Ctrl group in the current study, the contents of reproductive hormones and serotonin in the LA group were significantly decreased, indicating that gonadal hormone secretion was disordered. After the intervention of MSCs, reproductive hormones content and serotonin content increase, and gonadal hormone secretion tended to a normal level. Several researches stated that the decrease in T levels after exposure to LA toxicity may result from the indirect effects of LA on the hypothalamic-pituitary–testicular (HPT) axis, and the structural and functional integrity of male reproductive organs [53, 54]. In line with earlier findings, a recent study that examined the proteins involved in the synthesis of steroid hormones in mouse testes discovered that lead significantly lowers serum testosterone levels and disrupts enzyme activity and gene expression levels during the synthesis of male steroid hormones [54–56].

Malonaldehyde was used in different studies as an indicator for oxidative stress either by its evaluation in the blood or in the tested tissues [57–59]. Since tissue MDA is a byproduct of peroxidized polyunsaturated fatty acids (PUFA), it is an essential diagnostic metric for identifying oxidative stress. An increase in lipid peroxidation is suggested by the elevated MDA level. MDA levels in rat tissues have been shown to rise in response to pesticides like acetamiprid and heavy metals like lead [51, 60]. In the present study, MDA level increased in the LA group compared to the other two groups. These results were consistent with recent reports that lead increased the levels of lipid peroxide (LPO) and MDA, in testicular tissue [56]. A higher level of oxidative stress (OS) can harm the gonads by causing damage to the nuclear and mitochondrial DNA, as well as the sperm plasma membrane. This is associated with shorter telomere length, the production of 8-OHdG, and mitochondrial DNA fragmentation. Leydig cell activity generally declines as a result of this, affecting proteins including the steroidogenic acute regulatory protein (StAR), which controls mitochondrial cholesterol uptake [61, 62].

The current study's findings unequivocally demonstrated that rats given lead acetate had much lower levels of the antioxidants SOD, catalase, and Gpx. These results are consistent with previous study showed that rats exposed to lead had significantly lower levels of the antioxidant enzymes SOD and catalase activity in their testes [63]. In the current investigation, rats given MSCs plus LA had higher levels of SOD, catalase, and Gpx than rats given lead alone. MSCs can eliminate free radicals, lower the oxidation rate, and increase the expression of the heme oxygenase system, particularly SOD [47].

One of the most dangerous hazards of lead is that it influence the genetic through chromosomal abnormalities [13, 64] and micronucleus [65]. These genotoxic may involve direct DNA damage that affects chromatin stabilization [66] or engaging with repair procedures [67]. In this study, the sequence sizes ranged from 537 to 540 base pairs (bp). According to Simon et al. [38], the 16Sar and 16Sbr primers amplify a fragment between 500 and 650 bp, so the observed sizes fell within the expected range. The final alignment obtained was 540 bp in length, with 534 of those sites being conserved. This aligns with previous studies reporting that mitochondrial genes are highly conserved across many animal species. [68, 69].

The high P-distance (0.0031) found between ctrl group and LA group in the current research reflected the genetic effects of lead acetate. Recent study agreed with these findings and illustrated presence of highest P-distance value (0.002) between the lead acetate-treated mice and the control non-treated one [70]. According to multiple studies, mice exposed to LA at doses of 50, 100, 200, and 400 mg/kg body weight exhibited a significant increase in the incidence of chromosomal abnormalities in their bone marrow [71]. Furthermore, it was noted that sucking rats exposed to lead either subchronically (for nine days) or cutely (in a single intraperitoneal injection) experienced genome damage from low doses of lead acetate, which had no effect on the growth and development of the rats [72]. Consequently, other research linked the direct impact of lead on DNA structure and oxidative processes to the observed damaging effect of lead on mice’s DNA [73, 74]. Additionally, lead ions were found to impair DNA repair mechanisms and reduce the fidelity of DNA synthesis [13, 75].

The results of 16S rRNA sequences displayed that the P-distances between the Ctrl group and LA-MSCs group was 0.0019 which was smaller than that in the LA group. Thus, stem cells reduced the genotoxic effect of LA that may be due to the MSCs were described as multi-potent because of their ability for differentiation into a variety of different cells and tissue lineages [76]. Stem cells models considered as new tools for drug discovery and predictive toxicology due to their exclusive characteristics relevant to stemness, self-renewal and differentiation ability to various cell types [77].

The histological investigation in the present study showed that, compared to the Ctrl group, the LA group's seminiferous tubules displayed significant degenerative and necrotic alterations, including edema in the interstitial tissue and desquamation of some necrotic cells. Similar results were recorded in the previous study that stated male wistar rats exposed to LA exhibited a testicular parenchymal atrophy, various deteriorating histological abnormalities, as well as inhibition of spermatogenesis [78]. Furthermore, a recent study investigating the effects of subacute lead exposure in rats [79] revealed that testicular injuries included degeneration and depletion of germinal cells, alterations in Sertoli cells, reduced semen quality, changes in the shape and size of spermatozoa, and inflammatory responses.

Johnsen’s score was used to evaluate lead-induced regressive histological damage. The observed damage, including disrupted seminiferous tubules and a reduction in spermatogenic cells, as indicated by lower Johnsen’s scores, may be correlated with impaired spermatogenesis [80, 81].

In the current study, the significant increase of the average percentage of Caspase-3 antibody in the LA group indicated the harmful effect of the LA toxicity in the reproductive aspects of the toxicated animal. The current findings are similar to that was reported in the recent studies [82, 83]. It was stated that LA activates caspase-3 in spermatogenic cells, which triggers apoptosis, and it also causes DNA damage and apoptosis in spermatozoa [54]. As a result, lead exposure has lately been identified as a significant contributor to male infertility and testicular dysfunction [54, 84]. Male infertility results from excessive exposure to lead compounds, which lowers semen quality and reproductive capacity [84].

In the present study, KI67 antibody revealed an obvious decrease in its average percentage in the testicular tissue of LA group, while its percentage significantly increased in the LA-MSCs group after treatment by MSCs. Similar findings were reported in the previous studies that indicate the deleterious effect of lead toxicity was not limited to damage of the tissue but extend to decrease the Ki-67 expression that is responsible for cell proliferation [85, 86]. According to earlier research, Ki-67 expression is a prognostic indicator for a number of malignancies and is connected with cell proliferation essential for this malignancies [87]. The latter study shows that the cancer stem cell niche is diminished in human epithelial breast and colon cancer cells when Ki-67 is genetically disrupted. This provides evidence that the commencement of Ki-67 expression and cell proliferation are positively correlated with stem cells.

Finally, MSCs therapy has shown potential in treating medical conditions, specifically lead poisoning-induced male infertility. However, the clinical relevance and/or limitations of translating these findings to human therapy should be investigated further. Research indicates that MSCs injections may enhance germ cell differentiation and support the regeneration of gonadal tissue. Previous experimental studies in animal models of azoospermia have demonstrated promising outcomes using MSCs-based approaches. Although these therapies are not yet considered standard practice in human medicine, the encouraging results provide a hopeful outlook for their future application in routine clinical settings [88, 89].

It is suggested that MSCs treatment may reduce the risk of infertility, especially in men with occupational exposure to lead, such as workers in industries like battery manufacturing, construction (especially demolition or renovation), smelting, and radiator repair. Using MSCs to treat male infertility caused by lead poisoning is a promising area of regenerative medicine. However, several limitations exist in these aspects, such as most data are from preclinical animal models, meaning human trials are needed to confirm safety and efficacy. Additionally, there are ethical and regulatory barriers, as stem cell therapies require strict regulation, particularly regarding cell sourcing, purity, and delivery. Further optimization is needed, including determining the best type of MSCs (e.g., bone marrow, adipose tissue, umbilical cord), the optimal dose and administration route, and avoiding long-term effects and potential risks (e.g., tumorigenicity) [90, 91].

## Conclusion

The MSCs is a potential therapeutic for the treatment of LA related testicular dysfunction through the reduction of oxidative stress, reduction of the genotoxic effect, reduction of the apoptosis marker caspase-3, increase the proliferation marker Ki-67 in the testicular tissue and restoration of hormonal imbalance.

## Data Availability

This article contains all the data that was created or evaluated during the research.

## References

[1] Al-Mzaien AK, Khalaf OH. AL-Neamah GA, Al-Naimi RA: Study some of the histopathological changes of acute, subacute and chronic lead acetate toxicity related to catalase activity in blood of adult male Wistar rats. Kufa J Vet Med Sci. 2015;6(2):183–93.

[2] Abass MA, Refat NA. Efficacy of Myrrh extract"Mirazid (R){"} to reduce lead acetate toxicity in albino rats with special reference to cerebellum and testes. Life Sci J. 2011;8(4):406–14.

[3] Sansar W, Bouyatas MM, Ahboucha S, Gamrani H. Effects of chronic lead intoxication on rat serotoninergic system and anxiety behavior. Acta Histochem. 2012;114(1):41–5. 21392819 10.1016/j.acthis.2011.02.003

[4] Gurer H, Ercal N. Can antioxidants be beneficial in the treatment of lead poisoning? Free Radical Biol Med. 2000;29(10):927–45. 11084283 10.1016/s0891-5849(00)00413-5

[5] Lamidi I, Akefe I. Mitigate effects of antioxidants in lead toxicity. Clin Pharmacol Toxicol J. 2017;1(1):1–9.

[6] Baranowska-Bosiacka I, Gutowska I, Rybicka M, Nowacki P, Chlubek D. Neurotoxicity of lead. Hypothetical molecular mechanisms of synaptic function disorders. Neurol Neurochir Pol. 2012;46(6):569–78. 23319225 10.5114/ninp.2012.31607

[7] Guidotti T, McNamara J, Moses M. The interpretation of trace element analysis in body fluids. Indian J Med Res. 2008;128(4):524–32. 19106444

[8] Rastogi S. Renal effects of environmental and occupational lead exposure, vol. 12. Medknow; 2008. p. 103–6. 10.4103/0019-5278.44689 PMC2796746 20040966

[9] Pulido MD, Parrish AR. Metal-induced apoptosis: mechanisms. Mutation Res/Fundamental Mole Mech Mutagenesis. 2003;533(1–2):227–41. 10.1016/j.mrfmmm.2003.07.015 14643423

[10] Mabrouk A, Bel Hadj SI, Chaieb W, Ben Cheikh H. Protective effect of thymoquinone against lead-induced hepatic toxicity in rats. Environ Sci Pollut Res. 2016;23:12206–15. 10.1007/s11356-016-6419-5 26971798

[11] Morgan A, Ibrahim MA, Galal MK, Ogaly HA, Abd-Elsalam RM. Innovative perception on using Tiron to modulate the hepatotoxicity induced by titanium dioxide nanoparticles in male rats. Biomed Pharmacother. 2018;103:553–61. 29677542 10.1016/j.biopha.2018.04.064

[12] Sikka SC, Wang R. Endocrine disruptors and estrogenic effects on male reproductive axis. Asian J Androl. 2008;10(1):134–45. 18087652 10.1111/j.1745-7262.2008.00370.x

[13] Ahmed Y, Mahmoud G, Farghaly A, Abo-Zeid MA, Ismail E. Some studies on the toxic effects of prolonged lead exposure in male rabbits: chromosomal and testicular alterations. Global Veterinaria. 2012;8(4):360–6.

[14] Martin KK, Arsene A, Melaine M, Ernest Z, Joseph DA, Mireille D, David N. Effects of chronic lead exposure on zinc concentration and spermatic parameters in Wistar rats. Ann Med Biomed Sci. 2017;3(2):51–8.

[15] Thoreux-Manlay A, Le Goascogne C, Segretain D, Jégou B, Pinon-Lataillade G. Lead affects steroidogenesis in rat Leydig cells in vivo and in vitro. Toxicology. 1995;103(1):53–62. 8525490 10.1016/0300-483x(95)03107-q

[16] Biswas N, Ghosh P. Effect of lead on male gonadal activity in albino rats. Kathmandu Univ Med J (KUMJ). 2004;2(1):43–6. 19780287

[17] Hamadouche NA, Sadi N, Kharoubi O, Slimani M, Aoues A. The protective effect of vitamin E against genotoxicity of lead acetate intraperitoneal administration in male rat. Arch Biol Sci. 2013;65(4):1435–45.

[18] Queiroz EKRd, Waissmann W. Occupational exposure and effects on the male reproductive system. Cad Saude Publica. 2006;22:485–93. 16583092 10.1590/s0102-311x2006000300003

[19] Elgawish RAR, Abdelrazek HM. Effects of lead acetate on testicular function and caspase-3 expression with respect to the protective effect of cinnamon in albino rats. Toxicol Rep. 2014;1:795–801. 28962292 10.1016/j.toxrep.2014.10.010 PMC5598148

[20] Ahmed-Farid O, Hassan M. The protective effect of flaxseed oil supplemented with high source of branched chain amino acids against the rats testicular toxicity induced by lead acetate. World J Pharm Pharmaceutical Sci. 2017;6(12):30–42.

[21] Ramalho-Santos J, Amaral S, Oliveira PJ. Diabetes and the impairment of reproductive function: possible role of mitochondria and reactive oxygen species. Curr Diabetes Rev. 2008;4(1):46–54. 18220695 10.2174/157339908783502398

[22] Hu C, Wu Z, Li L. Mesenchymal stromal cells promote liver regeneration through regulation of immune cells. Int J Biol Sci. 2020;16(5):893. 32071558 10.7150/ijbs.39725 PMC7019139

[23] Zhang Y, Li R, Rong W, Han M, Cui C, Feng Z, Sun X, Jin S. Therapeutic effect of hepatocyte growth factor-overexpressing bone marrow-derived mesenchymal stem cells on CCl4-induced hepatocirrhosis. Cell Death Dis. 2018;9(12):1186. 30538216 10.1038/s41419-018-1239-9 PMC6290007

[24] Zargar MJ, Kaviani S, Vasei M, Soufi Zomorrod M, Heidari Keshel S, Soleimani M. Therapeutic role of mesenchymal stem cell-derived exosomes in respiratory disease. Stem Cell Res Ther. 2022;13(1):194. 35550188 10.1186/s13287-022-02866-4 PMC9096764

[25] Behnke J, Kremer S, Shahzad T, Chao C-M, Böttcher-Friebertshäuser E, Morty RE, Bellusci S, Ehrhardt H. MSC based therapies—new perspectives for the injured lung. J Clin Med. 2020;9(3):682. 32138309 10.3390/jcm9030682 PMC7141210

[26] Lee P-W, Wu B-S, Yang C-Y. Lee OK-S: Molecular mechanisms of mesenchymal stem cell-based therapy in acute kidney injury. Int J Mol Sci. 2021;22(21):11406. 34768837 10.3390/ijms222111406 PMC8583897

[27] Zou X, Jiang K, Puranik AS, Jordan KL, Tang H, Zhu X, Lerman LO. Targeting murine mesenchymal stem cells to kidney injury molecule-1 improves their therapeutic efficacy in chronic ischemic kidney injury. Stem Cells Transl Med. 2018;7(5):394–403. 29446551 10.1002/sctm.17-0186 PMC5905229

[28] Rezq A, Sameh B, Attar A, Elgazar A, Basalamah M. Protective effect of grape seeds powder against lead acetate-induced brain toxicity in male rats. World Appl Sci J. 2018;36(2):185–96.

[29] Reda S, Hashem H, Elnegris H, Elshal L. Role of bone marrow derived mesenchymal stem cells in protection of spermatogenic and Sertoli cells against histological alterations induced by torsion/detorsion in rats. Journal of medical histology. 2017;1(2):154–69.

[30] Mohammed SS, Mansour MF, Salem NA. Therapeutic effect of stem cells on male infertility in a rat model: histological, molecular, biochemical, and functional study. Stem Cells International. 2021;2021(1):8450721. 34733332 10.1155/2021/8450721 PMC8560298

[31] Yokoi K, Uthus EO, Nielsen FH. Nickel deficiency diminishes sperm quantity and movement in rats. Biol Trace Elem Res. 2003;93:141–53. 12835498 10.1385/BTER:93:1-3:141

[32] Sönmez M, Türk G, Yüce A. The effect of ascorbic acid supplementation on sperm quality, lipid peroxidation and testosterone levels of male Wistar rats. Theriogenology. 2005;63(7):2063–72. 15823361 10.1016/j.theriogenology.2004.10.003

[33] El-Maddawy ZK, El-Sayed YS. Comparative analysis of the protective effects of curcumin and N-acetyl cysteine against paracetamol-induced hepatic, renal, and testicular toxicity in Wistar rats. Environ Sci Pollut Res. 2018;25:3468–79. 10.1007/s11356-017-0750-3 29152699

[34] Ohkawa H, Ohishi N, Yagi K. Assay for lipid peroxides in animal tissues by thiobarbituric acid reaction. Anal Biochem. 1979;95(2):351–8. 36810 10.1016/0003-2697(79)90738-3

[35] Misra HP, Fridovich I. The role of superoxide anion in the autoxidation of epinephrine and a simple assay for superoxide dismutase. J Biol Chem. 1972;247(10):3170–5. 4623845

[36] Lück H. Catalase. In: Bergmeyer HU, editor. Methods of enzymatic analysis. Elsevier; 1963.

[37] Flohé L, Günzler WA. [12] Assays of glutathione peroxidase. In: Methods in enzymology, vol. 105. Elsevier; 1984. p. 114–20. 10.1016/s0076-6879(84)05015-1 6727659

[38] Simon C, Franke A, Martin A. The polymerase chain reaction: DNA extraction and amplification. In: Molecular techniques in taxonomy. Springer; 1991. p. 329–55.

[39] Edgar RC. MUSCLE: multiple sequence alignment with high accuracy and high throughput. Nucleic Acids Res. 2004;32(5):1792–7. 15034147 10.1093/nar/gkh340 PMC390337

[40] Tamura K, Stecher G, Kumar S. MEGA11: molecular evolutionary genetics analysis version 11. Mol Biol Evol. 2021;38(7):3022–7. 33892491 10.1093/molbev/msab120 PMC8233496

[41] Felsenstein J. Confidence limits on phylogenies: an approach using the bootstrap. Evolution. 1985;39(4):783–91. 28561359 10.1111/j.1558-5646.1985.tb00420.x

[42] Kimura M. A simple method for estimating evolutionary rates of base substitutions through comparative studies of nucleotide sequences. J Mol Evol. 1980;16:111–20. 7463489 10.1007/BF01731581

[43] Naeimi RA, Amiri FT, Khalatbary AR, Ghasemi A, Zargari M, Ghesemi M, Hosseinimehr SJ. Atorvastatin mitigates testicular injuries induced by ionizing radiation in mice. Reprod Toxicol. 2017;72:115–21. 28668617 10.1016/j.reprotox.2017.06.052

[44] Rashed M, Ragab N, Shalaby A, Ragab W. Patterns of testicular histopathology in men with primary infertility. Int J Urol. 2008;5:1–5.

[45] Adhikari N, Sinha N, Narayan R, Saxena D. Lead-induced cell death in testes of young rats. J Appl Toxicol: Int J. 2001;21(4):275–7. 10.1002/jat.754 11481659

[46] Richburg JH. The relevance of spontaneous-and chemically-induced alterations in testicular germ cell apoptosis to toxicology. Toxicol Lett. 2000;112:79–86. 10720715 10.1016/s0378-4274(99)00253-2

[47] Zhao S, Li Z, Li K, Dai X, Xu Z, Li L, Wang H, Liu X, Li D. repairing effect of mesenchymal stem cells on lead acetate-induced testicular injury in mice. Cell Transplant. 2024;33:09636897231219395. 38173262 10.1177/09636897231219395 PMC10768580

[48] Marzec-Wróblewska U, Kamiński P, Łakota P, Szymański M, Wasilow K, Ludwikowski G, Jerzak L, Stuczyński T, Woźniak A, Buciński A. Human sperm characteristics with regard to cobalt, chromium, and lead in semen and activity of catalase in seminal plasma. Biol Trace Elem Res. 2019;188:251–60. 29959647 10.1007/s12011-018-1416-9

[49] Sukhn C, Awwad J, Ghantous A, Zaatari G. Associations of semen quality with non-essential heavy metals in blood and seminal fluid: data from the environment and male infertility (EMI) study in Lebanon. J Assist Reprod Genet. 2018;35:1691–701. 29931406 10.1007/s10815-018-1236-z PMC6133818

[50] Amin YA, Noseer EA, Fouad SS, Ali RA, Mahmoud HY. Changes of reproductive indices of the testis due to Trypanosoma evansi infection in dromedary bulls (Camelus dromedarius): semen picture, hormonal profile, histopathology, oxidative parameters, and hematobiochemical profile. J Adv Vet Animal Res. 2020;7(3):537. 10.5455/javar.2020.g451 PMC7521820 33005681

[51] Toghan R, Amin YA, Ali RA, Fouad SS. Ahmed MA-EB, Saleh SM: protective effects of Folic acid against reproductive, hematological, hepatic, and renal toxicity induced by Acetamiprid in male Albino rats. Toxicology. 2022;469: 153115. 35124148 10.1016/j.tox.2022.153115

[52] Ali RA, Awadalla EA, Amin YA, Fouad SS. Ahmed MA-EB, Hassan MH, Abdel-Kahaar E, Abdel-Aziz RH: The deleterious effects of sofosbuvir and ribavirin (antiviral drugs against hepatitis C virus) on different body systems in male albino rats regarding reproductive, hematological, biochemical, hepatic, and renal profiles and histopathological changes. Sci Rep. 2024;14(1):5682. 38453980 10.1038/s41598-024-55950-5 PMC10920821

[53] Anjum MR, Reddy PS. Recovery of lead-induced suppressed reproduction in male rats by testosterone. Andrologia. 2015;47(5):560–7. 24909355 10.1111/and.12303

[54] Hassan E, El-Neweshy M, Hassan M, Noreldin A. Thymoquinone attenuates testicular and spermotoxicity following subchronic lead exposure in male rats: Possible mechanisms are involved. Life Sci. 2019;230:132–40. 31136753 10.1016/j.lfs.2019.05.067

[55] Abdelhamid FM, Mahgoub HA, Ateya AI. Ameliorative effect of curcumin against lead acetate–induced hemato-biochemical alterations, hepatotoxicity, and testicular oxidative damage in rats. Environ Sci Pollut Res. 2020;27:10950–65. 10.1007/s11356-020-07718-3 31953765

[56] Zhao Z-M, Mei S, Zheng Q-Y, Wang J, Yin Y-R, Zhang J-J, Wang X-Z. Melatonin or vitamin C attenuates lead acetate-induced testicular oxidative and inflammatory damage in mice by inhibiting oxidative stress mediated NF-κB signaling. Ecotoxicol Environ Saf. 2023;264: 115481. 37716076 10.1016/j.ecoenv.2023.115481

[57] Amin YA, Omran GA, Fouad SS, Fawy MA, Ibrahim RM, Khalifa FA, Ali RA. Abortion associated with postpartum opportunistic bacterial invasion reduces fertility and induces disturbances of reproductive hormones, hematological profile, and oxidant/antioxidant profiles in dairy cows. J Adv Vet Animal Res. 2023;10(4):654. 10.5455/javar.2023.j721 PMC10868696 38370890

[58] El-Sawy SA, Amin YA, El-Naggar SA, Abdelsadik A. Artemisia annua L.(Sweet wormwood) leaf extract attenuates high-fat diet-induced testicular dysfunctions and improves spermatogenesis in obese rats. J Ethnopharmacol. 2023;313:116528. 37127141 10.1016/j.jep.2023.116528

[59] Amin YA, Ali RA, Fouad SS, Ibrahim RM. The deleterious effect of postpartum pyometra on the reproductive indices, the metabolic profile, and oxidant/antioxidant parameters of dairy cows. Vet World. 2021;14(2):329. 33776298 10.14202/vetworld.2021.329-338 PMC7994124

[60] Bas H, Kalender S. Antioxidant status, lipid peroxidation and testis-histoarchitecture induced by lead nitrate and mercury chloride in male rats. Braz Arch Biol Technol. 2016;59: e16160151.

[61] Mohlala K, Offor U, Monageng E, Takalani NB, Opuwari CS. Overview of the effects of moringa oleifera leaf extract on oxidative stress and male infertility: a review. Appl Sci. 2023;13(7):4387.

[62] Terasaka T, Adakama ME, Li S, Kim T, Terasaka E, Li D, Lawson MA. Reactive oxygen species link gonadotropin-releasing hormone receptor signaling cascades in the gonadotrope. Front Endocrinol. 2017;8:286. 10.3389/fendo.2017.00286 PMC5671645 29163358

[63] Anjum MR, Reddy P. Effect of perinatal exposure to lead acetate on testicular lipid peroxidation in adult rats. Int J Pharm Bio Sci. 2013;4(1):893–8.

[64] García-Lestón J, Méndez J, Pásaro E, Laffon B. Genotoxic effects of lead: an updated review. Environ Int. 2010;36(6):623–36. 20466424 10.1016/j.envint.2010.04.011

[65] Celik A, Öğenler O, Çömelekoğlu Ü. The evaluation of micronucleus frequency by acridine orange fluorescent staining in peripheral blood of rats treated with lead acetate. Mutagenesis. 2005;20(6):411–5. 16135535 10.1093/mutage/gei055

[66] Johansson L, Pellicciari CE. Lead-induced changes in the stabilization of the mouse sperm chromatin. Toxicology. 1988;51(1):11–24. 3413797 10.1016/0300-483x(88)90076-5

[67] Hartwig A, Schlepegrell R, Beyersmann D. Indirect mechanism of lead-induced genotoxicity in cultured mammalian cells. Mutation Res/Genetic Toxicol. 1990;241(1):75–82. 10.1016/0165-1218(90)90110-n 2333087

[68] van der Kuyl AC, Kuiken CL, Dekker JT, Goudsmit J. Phylogeny of African monkeys based upon mitochondrial 12S rRNA sequences. J Mol Evol. 1995;40:173–80. 7535363 10.1007/BF00167111

[69] Saikia DP, Kalita DJ, Borah P, Sarma S, Dutta R, Rajkhowa D. Molecular characterization of the mitochondrial 16S rRNA gene of cattle, buffalo and yak. Veterinarski Arhiv. 2016;86(6):777–85.

[70] Allam M, Said A, Mar’ie Z. Impact of titanium dioxide nanoparticles on lead acetate-induced genotoxicity in the major histocompatibility complex region and 16S rRNA sequence in mice. SVU-Int J Agric Sci. 2021;3(4):50–62.

[71] Fahmy MA. Lead acetate genotoxicity in mice. Cytologia. 1999;64(4):357–65.

[72] Kašuba V, Rozgaj R, Fučic A, Varnai VM, Piasek M. Lead acetate genotoxicity in suckling rats. Biologia Bratislava. 2004;59(6):779–85.

[73] Stohs SJ, Bagchi D. Oxidative mechanisms in the toxicity of metal ions. Free Radical Biol Med. 1995;18(2):321–36. 7744317 10.1016/0891-5849(94)00159-h

[74] Gargioni R, Neto FF, Buchi D, Randi M, Franco C, Paludo K, Pelletier E, Ferraro M, Cestari M, Bussolaro D. Cell death and DNA damage in peritoneal macrophages of mice (Mus musculus) exposed to inorganic lead. Cell Biol Int. 2006;30(7):615–23. 16757190 10.1016/j.cellbi.2006.03.010

[75] Acharya U, Acharya S, Mishra M. Lead acetate induced cytotoxicity in male germinal cells of Swiss mice. Ind Health. 2003;41(3):291–4. 12916762 10.2486/indhealth.41.291

[76] Ahmed SK, Mohammed SA, Khalaf G, Fikry H. Role of bone marrow mesenchymal stem cells in the treatment of CCL4 induced liver fibrosis in albino rats: a histological and immunohistochemical study. Int J Stem Cells. 2014;7(2):87–97. 25473446 10.15283/ijsc.2014.7.2.87 PMC4249908

[77] Rezvanfar MA, Hodjat M, Abdollahi M. Growing knowledge of using embryonic stem cells as a novel tool in developmental risk assessment of environmental toxicants. Life Sci. 2016;158:137–60. 27208651 10.1016/j.lfs.2016.05.027

[78] Dorostghoal M, Seyyednejad SM, Nejad MNT. Cichorium intybus L extract ameliorates testicular oxidative stress induced by lead acetate in male rats. Clin Exp Reprod Med. 2020;47(3):161. 32862634 10.5653/cerm.2019.03496 PMC7482945

[79] Solcan C, Şlencu BG, Trinca LC, Motrescu I, Petrovici A, Solcan G: Testicular injuries due to subacute combined exposure to cadmium and lead in rats. *Farmacia* 2022; 70(4).

[80] Mohammadi S, Gholamin M, Mohammadi M, Mansouri A, Mahmoodian R, Attari S, Kebriaei S, Zibaei B, Roshanaei M, Daneshvar F. Down-regulation of CatSper 1 and CatSper 2 genes by lead and mercury. Environ Toxicol Pharmacol. 2018;59:82–6. 29549816 10.1016/j.etap.2018.03.007

[81] Rao F, Zhai Y, Sun F. Punicalagin mollifies lead acetate-induced oxidative imbalance in male reproductive system. Int J Mol Sci. 2016;17(8):1269. 27529221 10.3390/ijms17081269 PMC5000667

[82] Tuncer SÇ, Akarsu SA, Küçükler S, Gür C, Kandemir FM. Effects of sinapic acid on lead acetate-induced oxidative stress, apoptosis and inflammation in testicular tissue. Environ Toxicol. 2023;38(11):2656–67. 37471654 10.1002/tox.23900

[83] Akarsu SA, Gür C, İleritürk M, Akaras N, Küçükler S, Kandemir FM. Effect of syringic acid on oxidative stress, autophagy, apoptosis, inflammation pathways against testicular damage induced by lead acetate. J Trace Elem Med Biol. 2023;80: 127315. 37801787 10.1016/j.jtemb.2023.127315

[84] Li C, Zhao K, Zhang H, Liu L, Xiong F, Wang K, Chen B. Lead exposure reduces sperm quality and DNA integrity in mice. Environ Toxicol. 2018;33(5):594–602. 29446210 10.1002/tox.22545

[85] Falana B, Ogundele O, Duru F, Oshinubi A, Falode D. Role of Se+ Zn in regeneration (Ki-67) following Pb toxicity (p53andcad) in the germinal epithelium of adult Wistar rats. Pakistan J Biol Sci: PJBS. 2013;16(2):67–73. 10.3923/pjbs.2013.67.73 24199489

[86] Ainehchi N, Khaki A, Merat E. Ocimum basilicum extract ameliorate lead-induced testicular apoptosis in rats. Afr J Tradit Complement Altern Med. 1998;80:98–103.

[87] Cidado J, Wong H, Rosen D, Cimino-Mathews A, Garay J, Fessler A, Rasheed Z, Hicks J, Cochran R, Croessmann S. Ki-67 is required for maintenance of cancer stem cells but not cell proliferation. Oncotarget. 2016;7:6281–93. 26823390 10.18632/oncotarget.7057 PMC4868756

[88] Zhankina R, Baghban N, Askarov M, Saipiyeva D, Ibragimov A, Kadirova B, Khoradmehr A, Nabipour I, Shirazi R, Zhanbyrbekuly U. Mesenchymal stromal/stem cells and their exosomes for restoration of spermatogenesis in non-obstructive azoospermia: a systemic review. Stem Cell Res Ther. 2021;12:1–12. 33823925 10.1186/s13287-021-02295-9 PMC8025392

[89] Zhankina R, Zare A, Afshar A, Khoradmehr A, Dorvash MR, Rahmanifar F, Tanideh N, Koohi Hosseinabadi O, Arabi Monfared A, Zare S: Extracellular vesicles present in bone marrow mesenchymal stromal/stem cell con-ditioned media restore spermatogenesis in azoospermic mice. Int J Fertility Sterility 2024. 10.22074/IJFS.2024.2021445.1619 PMC12207096 40590291

[90] Cassim MI, Mohamed T. A novel therapy for the treatment of malefactor infertility due to non-obstructive azoospermia: a case report. Crescent J Med Biol Sci. 2019;6(1):129–31.

[91] Zhankina R, Zhanbyrbekuly U, Askarov M, Zare A, Jafari N, Saipiyeva D, Sherkhanov R, Akhmetov D, Hashemi A, Farjam M. Improving fertility in non-obstructive azoospermia: results from an autologous bone marrow-derived mesenchymal stromal/stem cell phase i clinical trial. Int J Fertility Sterility. 2024;18(Suppl 1):60. 10.22074/IJFS.2023.2005045.1480 PMC11263852 39033372

